# A genome-wide association study of intra-ocular pressure suggests a novel association in the gene *FAM125B* in the TwinsUK cohort

**DOI:** 10.1093/hmg/ddu050

**Published:** 2014-02-11

**Authors:** Abhishek Nag, Cristina Venturini, Kerrin S. Small, International Glaucoma Genetics Consortium, Terri L. Young, Ananth C. Viswanathan, David A. Mackey, Pirro G. Hysi, Christopher Hammond, Tin Aung, Ching-Yu Cheng, Brian W Fleck, Jane Gibson, Alex W Hewitt, Albert Hofman, René Höhn, Jost B Jonas, Chiea-Cheun Khor, Caroline CW Klaver, Hans G Lemij, Jiemin Liao, Andrew J Lotery, Yi Lu, Stuart Macgregor, Paul Mitchell, Wishal D Ramdas, Henriët Springelkamp, E-Shyong Tai, Yik-Ying Teo, André G Uitterlinden, Cornelia M van Duijn, Leonieke ME van Koolwijk, Johannes R Vingerling, Veronique Vitart, Eranga Vithana, Jie Jin Wang, Katie M Williams, Robert Wojciechowski, Tien-Yin Wong, WTCCC2, Liang Xu, Ekaterina Yonova-Doing, Zeller Tanja

**Affiliations:** 1 Department of Twin Research and Genetic Epidemiology, King's College London, St. Thomas’ Hospital, London, UK; 2 UCL Institute of Ophthalmology, London, UK; 3 Center for Human Genetics, Duke University Medical Center, Durham, NC, USA; 4 NIHR Biomedical Research Centre at Moorfields Eye Hospital NHSFT and UCL Institute of Ophthalmology, London, UK; 5 Centre for Ophthalmology and Visual Science, Lions Eye Institute, University of Western Australia, Perth, Australia

## Abstract

Glaucoma is a major cause of blindness in the world. To date, common genetic variants associated with glaucoma only explain a small proportion of its heritability. We performed a genome-wide association study of intra-ocular pressure (IOP), an underlying endophenotype for glaucoma. The discovery phase of the study was carried out in the TwinsUK cohort (*N* = 2774) analyzing association between IOP and single nucleotide polymorphisms (SNPs) imputed to HapMap2. The results were validated in 12 independent replication cohorts of European ancestry (combined *N* = 22 789) that were a part of the International Glaucoma Genetics Consortium. Expression quantitative trait locus (eQTL) analyses of the significantly associated SNPs were performed using data from the Multiple Tissue Human Expression Resource (MuTHER) Study. In the TwinsUK cohort, IOP was significantly associated with a number of SNPs at 9q33.3 (*P* = 3.48 × 10^−8^ for rs2286885, the most significantly associated SNP at this locus), within the genomic sequence of the *FAM125B* gene. Independent replication in a composite panel of 12 cohorts revealed consistent direction of effect and significant association (*P* = 0.003, for fixed-effect meta-analysis). Suggestive evidence for an eQTL effect of rs2286885 was observed for one of the probes targeting the coding region of the *FAM125B* gene. This gene codes for a component of a membrane complex involved in vesicular trafficking process, a function similar to that of the Caveolin genes (*CAV1* and *CAV2*) which have previously been associated with primary open-angle glaucoma. This study suggests a novel association between SNPs in *FAM125B* and IOP in the TwinsUK cohort, though further studies to elucidate the functional role of this gene in glaucoma are necessary.

## INTRODUCTION

Glaucoma represents a group of optic neuropathies characterized by a typical pattern of optic nerve damage resulting from loss of retinal ganglion cells and axons. Glaucoma accounts for a significant portion of the global burden of visual impairment, being the third leading cause of visual impairment and the second leading cause of blindness in the world (1).

Raised intra-ocular pressure (IOP) is a major modifiable risk factor for glaucoma (2,3) and treatment directed at lowering IOP remains the mainstay of current glaucoma treatment (3,4). IOP is a heritable trait, with heritability estimates ranging from 0.35 to 0.74 in different studies (5–7). Investigating genetic variation influencing IOP might help better understand IOP regulation and its role in glaucoma pathophysiology. So far, in the case of glaucoma, case–control studies and quantitative trait-based approaches have jointly identified a number of genes that influence the risk of developing clinical glaucoma as well as cause incremental changes in population-wide risk of the underlying quantitative traits. These include the association of *TMCO1* with open-angle glaucoma (OAG) (8) and IOP (9), and the association of *CDKN2B* with OAG (8,10) and vertical cup-to-disc ratio (11). This convergence of evidence between variants associated with glaucoma and those associated with ‘healthy’ variation in quantitative traits underlying the disease, justifies the use of quantitative traits (endophenotypes) to dissect the genetic basis of glaucoma.

Here, we describe a genome-wide association study (GWAS) investigating the healthy variation of IOP in general populations of European ancestry and report findings about genetic variants that we found consistently associated with IOP in the participating cohorts.

## RESULTS

Association testing in the TwinsUK cohort was performed for ∼1.87 million SNPs that passed our quality control (QC) criteria. Both the genomic inflation factor (1.036) and the quantile plot **(**Supplementary material, Fig. S1**)** of the results indicate no significant inflation of test statistics due to population stratification.

**Figure 1.**
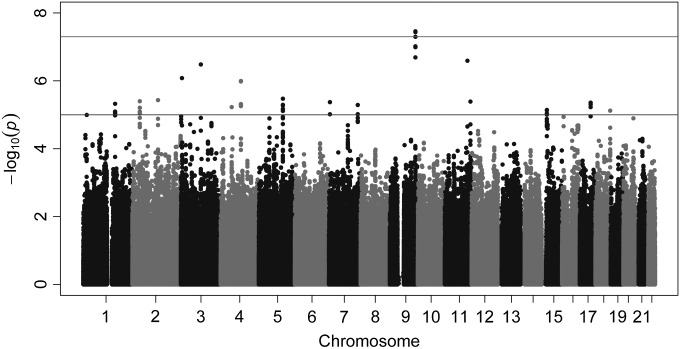
Results of the GWAS in the TwinsUK cohort (The upper line in the plot demarcates SNPs that are genome-wide significant (*P* < 5 × 10^−8^) and the lower line demarcates SNPs that show suggestive significance (*P* < 5 × 10^−6^)).

**Figure 2.**
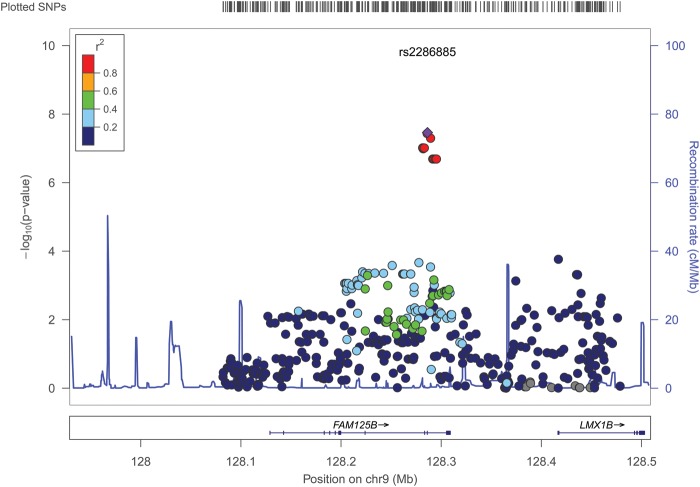
Regional association plot of the region 9q33.3 in the Twins UK cohort.

The region most significantly associated with IOP was 9q33.3, where three SNPs crossed the conventional threshold for genome-wide significance (Figs 1 and 2). In no other region SNPs crossed this significance threshold. Of the two previously reported IOP loci by van Koolwijk *et al.* (9), in which TwinsUK was one of the participating studies, rs11656696 on the *GAS7* locus showed statistically significant replication (*P* = 1.36 × 10^−2^), although not at a GWAS level; while the association for rs7555523 on the *TMCO1* locus was not statistically significant (*P* = 2.4 × 10^−1^) (9).

The most significantly associated SNP at 9q33.3 was rs2286885 (*P* = 3.48 × 10^−8^). Each copy of the A allele of this SNP increased IOP on average by 0.56 mmHg (95% CI: 0.36–0.75 mmHg). Two other SNPs at this locus that were significantly associated with IOP (rs2286886 and rs10819169) were in perfect linkage disequilibrium (LD) with rs2286885. This association remained the same (*β* = 0.56, SE = 0.103, *P* = 6.5 × 10^−8^ for rs2286885) after inclusion of the genotyping batch as a covariate in the analysis. For rs2286885, adjustment for central corneal thickness (CCT) improved the evidence for its association (*β* on adjustment for CCT = 0.59 mmHg; *P* = 1.16 × 10^−9^); adjustment for SBP, on the other hand, decreased the significance of association (*β* on adjustment for SBP = 0.50 mmHg; *P* = 6.46 × 10^−7^). The direction and magnitude of effect for rs2286885 remained unchanged on stratifying by sex and after selecting one twin from each pair.

The rs2286885 polymorphism is located within intron 8 of the *FAM125B* gene (*Homo sapiens* family with sequence similarity 125, member B)*. FAM125B* codes for two different isoforms (isoform 1 and isoform 2) that contain 10 and 6 exons, respectively. The protein encoded by this gene (multivesicular body subunit 12B, also known as MVB12B) is a component of the ESCRT-I (endosomal sorting complex required for transport I) complex, a heterotetramer, which mediates the sorting of ubiquitinated cargo proteins from the plasma membrane to the endosomal vesicle.

**Figure 3.**
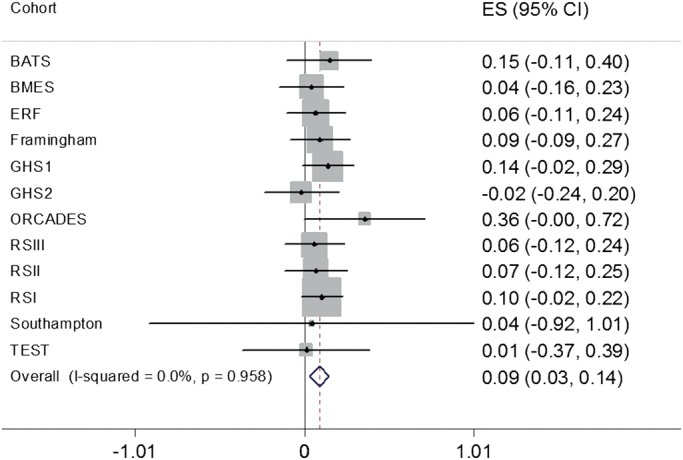
Forest plot for rs2286885 for the replication cohorts. ES, effect size; CI, confidence intervals; BATS, Brisbane Adolescent Twin Study; BMES, Blue Mountains Eye Study; ERF, Erasmus Rucphen Family Study; GHS, Gutenberg Health Study; ORCADES, Orkney Complex Disease Study; RS, Rotterdam Study; TEST, Tasmanian Eye Study of Twins.

We attempted to replicate the most significantly associated SNP at 9q33.3, rs2286885, in European cohorts participating in the International Glaucoma Genetics Consortium (IGGC). With the exception of one cohort, the direction of effect for rs2286885 was consistent with the one observed in the TwinsUK in all the other replication cohorts (Table 1). In a combined fixed-effect inverse variance meta-analysis of all the replication cohorts, rs2286885 was associated with IOP (*P* = 0.003) (Fig. 3). The magnitude of the effect was noticeably less in the replication panel, with each copy of the risk allele (A) of rs2286885 increasing IOP by 0.09 mmHg (95% CI: 0.03–0.14 mmHg). A joint meta-analysis of the discovery and replication samples thus reduced the strength of association (*P* = 5.67 × 10^−6^), with each copy of the risk allele (A) of rs2286885 increasing IOP by 0.12 mmHg (95% CI: 0.07–0.18 mmHg).

**Table 1.** Results for rs2286885 in the discovery and replication studies

Cohort	Number of subjects^a^	Frequency^b^	*β*^b^	SE	*P*-value
TwinsUK (discovery study)	2772	0.55	0.56	0.101	3.48 × 10^−8^
BATS	1152	0.53	0.147	0.13	0.2602
BMES	1667	0.55	0.0384	0.099	0.6994
ERF	2589	0.57	0.064	0.089	0.476
Framingham	2455	0.54	0.090	0.091	0.327
GHS1	2727	0.56	0.136	0.078	0.081
GHS2	1130	0.55	−0.020	0.113	0.858
ORCADES	474	0.54	0.358	0.184	0.051
RSIII	2034	0.57	0.057	0.092	0.537
RSII	2116	0.56	0.066	0.094	0.483
RSI	5782	0.57	0.101	0.062	0.106
Southampton	166	0.55	0.042	0.492	0.932
TEST	663	0.53	0.010	0.194	0.960

BATS, Brisbane Adolescent Twin Study; BMES, Blue Mountains Eye Study; ERF, Erasmus Rucphen Family Study; GHS, Guttenberg Health Study; ORCADES, Orkney Complex Disease Study; RS, Rotterdam Study; TEST, Tasmanian Eye Study of Twins; SE, dtandard error.

^a^Number of subjects refers to those with genotype information for rs2286885.

^b^Frequency and beta refer to the frequency and the effect size respectively for the A allele of rs2286885.

We checked for associations between rs2286885 and the three probes of the HumanHT-12 array covering *FAM125B* coding regions. There was some evidence for an eQTL association, with the best observed SNP-expression association being between rs2286885 (which was the top IOP-associated SNP at the locus) and the probe (ILMN_1652525) targeting the isoform 1 of *FAM125B* in fat tissue (*P* = 0.02). The observed eQTL association however does not survive multiple testing corrections for number of probes and number of tissues tested.

## DISCUSSION

Here we report a novel association between variants located on the long arm of chromosome 9 and IOP levels. Our conclusions were based on GWAS results of one population of European descent, and replication of the variants in a compound panel of 12 independent populations of the same ancestry. The SNPs at 9q33.3 associated with IOP are within the LD block that contains the gene *FAM125B*. The protein product of *FAM125B* forms a component of ESCRT-I, a highly conserved complex (12), that is involved in vesicular trafficking process (also known as transcytosis). It is thought that vesicular transport/cell membrane remodelling pathways in the endothelial cells of the eye regulate IOP through active modulation of the formation of giant vacuoles and endothelial pores (13), which controls the drainage rather than the production of aqueous humour in the eye. Further support for the importance of this metabolic pathway in the determination of IOP is the recent discovery of the formation of intracellular vesicles in cultured human trabeculocytes in response to treatment with latanoprost, a drug which is a well known first line drop treatment to lower IOP (14). Incidentally, a recent study has identified association for IOP with variants in another gene (*ICA1)* that is also involved in vesicular transportation/cell membrane remodelling (15). In the absence of a case–control panel to test the role of the associated SNPs in *FAM125B* in glaucoma, we hypothesize that *FAM125B* could potentially mediate susceptibility to glaucoma through the vesicular transportation/cell membrane remodelling pathways, which have previously been implicated in glaucoma through the reported association of the *CAV1–CAV2* genes (16).

*FAM125B* mRNA is highly expressed in the human retina (http://biogps.org/). The result of our eQTL analysis was constrained by tissue availability. We observed only modest eQTL association for rs2286885 and *FAM125B* in one of the three non-ocular tissues we studied; however the possibility of existence of such an effect cannot be ruled out as gene expression is known to be a tissue-specific phenomenon (17).

Our results are important for a number of reasons. First they are illustrative of the fact that the path towards elucidating the genetic architecture of the complex traits such as IOP is still long and that there is still place for GWAS to identify genes and genetically controlled mechanisms that had previously eluded us. The effect size of the variants identified in *FAM125B* is small, as is the portion of the phenotypic variance that can be attributed to them. The findings are consistent with GWAS findings for most complex diseases to date (18), which has changed the way we look at complex disease genetics from an oligogenic to a plurigenic model, with hundreds and potentially thousands of individual variants contributing to phenotypic variation (19).

Second, our results offer an interesting insight on the phenomenon of ‘winner's curse’. Selection of SNP associations, robust to conservative Bonferroni correction, to be taken forward to subsequent replication stages, often leads to a bias towards selecting SNPs whose effect sizes in the discovery cohort are over-estimated, albeit truly positive (20). This way of selecting replication SNPs encourages a ‘winner's curse’ phenomenon. This is neither a new nor a harmful phenomenon; it has been well documented for some of the major confirmed results for a variety of traits in the GWAS era (21). Thus, in the discovery phase of our study, the estimated effect size of the associated variants at 9q33.3 appears to be over-estimated; while the effect sizes observed in the replication cohorts might reflect the true effect of the variants.

Third, these results show the wide range of possibilities that can explain the connection between DNA sequence variations with phenotypic expression. The associated variants in *FAM125B* were located within the intron 8 of the gene. The exons that flank this intron code for the isoform 1 of the protein product; on the other hand, the flanking exons are spliced out of the pre-mRNA when coding for the isoform 2. Moreover, our eQTL results showed association with only the isoform 1 of the gene, but not with the isoform 2. Our results could suggest a possible link between alternative splicing/ isoform expression at this locus and IOP regulation, but tissue specificity is likely to remain a difficult problem to tackle when studying expression of genes involved in ocular phenotypes.

Fourth, it is known that CCT influences IOP readings (22,23), and with CCT being a highly heritable trait (estimates range from 0.72 to 0.95) (5,6,24), there exists the possibility of CCT confounding genetic associations for IOP. Adjustment for CCT in our analysis improved the strength of association for SNPs at 9q33.3 locus, thus highlighting the fact that the locus regulates IOP rather than CCT. This also supports the notion that phenotype refinement for complex traits offers an effective strategy to improve the power to detect genetic variants underlying them.

A potential drawback of our study is that the discovery phase was conducted in a cohort with specific demographic features—a UK-based, predominantly female population. This might limit generalization, so replication of our findings in other cohorts with differing demographic structures is important. Three out of the four Asian cohorts of IGGC did not replicate the direction of effect for rs2286885 (Supplementary Material, Table S2**)**, a finding that could reflect differences in LD pattern at this locus between European and Asian populations, justifying our decision to include only the European cohorts in the replication. We further investigated the population diversity of the associated variants at 9q33.3 in European (HapMap-CEU) and Asian (HapMap-HCB and HapMap-JPT) population panels of HapMap. A significant difference in the allele frequency pattern of rs2286885 was noted between European and Asian populations—the frequency of the risk allele (A) of this SNP was about 55% in the European population, while it was 25 and 28% in the two Asian populations. This is indicative of differing haplotypic frequencies, and thus differences in the patterns of LD at this locus between European and Asians populations. Such variations in LD patterns between populations, if not accounted for, can confound the ability to detect associations when analyzing ethnically heterogenous population panels (25).

Although GWAS evidence presented here suggests association between *FAM125B* and IOP, short of any direct biological evidence, association results remain probabilistic that still have the potential of a type I error. Results from individual GWAS may be opening small windows into the genetic architecture of IOP and, by extension, glaucoma; however, functional characterization of the gene function will be necessary to fulfil the potential translational benefits of such studies.

## MATERIALS AND METHODS

We analysed 2774 participants (95% female and all of Caucasian ancestry) within the TwinsUK adult twin registry based at St. Thomas’ Hospital in London (26) for whom both genotype and IOP information was available (Supplementary Material, Table S1). Of the 2774 subjects, 681 were monozygotic twin pairs, 627 were dizygotic twin pairs and 158 were unrelated individuals. Twins largely volunteered unaware of the eye studies interests at the time of enrolment and gave fully informed consent under a protocol reviewed by the St. Thomas’ Hospital Local Research Ethics Committee. Exclusion criteria included any form of glaucoma surgery such as trabeculectomy or laser surgery that could alter IOP.

We measured IOP with a non-contact air-puff tonometer. The Ocular Response Analyser (ORA; Reichert^®^, Buffalo, NY, USA) ejects an air impulse in order to flatten the cornea, which is detected by an electro-optical collimation system. The mean IOP was calculated from four readings (two from each eye) for each participant. IOP for subjects receiving IOP-lowering medications (26 out of 2774) was imputed by increasing the measured value by 30%, based on efficacy data from commonly prescribed therapies (27). As CCT and systolic blood pressure (SBP) are known to influence IOP measurements (23), they were included as covariates in the analysis. CCT was measured using an ultrasound pachymetry device provided with the ORA instrument. Three SBP measurements were made for each subject using automated calibrated instrument, of which the average of the second and the third readings were considered for the analysis.

Subjects were genotyped in two different batches of approximately the same size, using two genotyping platforms from Illumina: 300 K Duo and HumanHap610-Quad arrays. Whole genome imputation of the genotypes was performed using HapMap2 (www.hapmap.org) haplotypes.

Stringent QC measures were implemented, including minimum genotyping success rate (>95%), Hardy–Weinberg equilibrium (*P* > 10^−6^), minimum MAF (>1%) and imputation quality score (>0.7). Subjects of non-Caucasian ancestry were excluded from the analysis.

A linear regression model, adjusted for age and sex, was fitted to test for association between genome-wide SNPs as independent and IOP as the dependent variable. An additive model of effect for the risk allele of an SNP was implemented. Additional analyses were performed with CCT and SBP as covariates in the analysis. A score test statistic as implemented in MERLIN (28), that takes into account the pedigree structure and the zygosity of the twins, was used to adjust for the non-independence of the observations.

Loci conventionally considered genome-wide significant (*P* < 5 × 10^−8^) in the discovery cohort were taken forward and meta-analysed using the summary statistics data obtained from 12 independent cohorts of European ancestry that were a part of the IGGC. Individual cohorts performed the replication association analyses for rs2286885 based on a protocol decided by the consortium in order to ensure consistency in the analyses. The replication cohorts had a combined sample size of 22 789; complete descriptions of the study populations, phenotyping and genotyping methods for the replication studies are provided in the Supplementary Material. Since differences in LD patterns between populations are known to affect the portability of phenotypic associations when the replication effort is attempted in populations that are distinct from the original population in which the genome-wide study is performed (25), four cohorts of Asian ancestry that were also a part of the IGGC were not included in our replication analysis. Where more than one SNP at a locus was genome-wide significant, i.e. in presence of LD, only the single most associated SNP was chosen for replication. A fixed-effect inverse variance meta-analysis of all the cohorts was performed using the module ‘metan’ on Stata Statistical Software, 11 (College Station, TX, USA).

Gene expression data for a subset of the TwinsUK cohort was obtained from the MuTHER study (29). As a part of the MuTHER study, 855 subjects from the TwinsUK cohort had their transcript expression quantified in three different tissue types (skin, fat and LCLs). This was done for 18 170 genes across the genome using 27 499 probes on Illumina's whole genome expression array (HumanHT-12 version 3) further details of the study methods are available in Nica *et al.* (29). For the genome-wide significant SNPs in the TwinsUK cohort, we tested for a possible eQTL effect on the overlapping gene.

## SUPPLEMENTARY MATERIAL

Supplementary Material is available at *HMG* online.

## FUNDING

The study was funded by the Wellcome Trust (grant WT081878MA), Guide Dogs for the Blind, and the European Community's Seventh Framework Programme (FP7/2007–2013). SNP Genotyping was performed by National Eye Institute via NIH/CIDR (grant R01EY018246-01-1, PI Young TL) and the Wellcome Trust Sanger Institute. The study also receives support from the National Institute for Health Research (NIHR)-funded BioResource, Clinical Research Facility and Biomedical Research Centre based at Guy's and St Thomas' NHS Foundation Trust in partnership with King's College London. A.N. received funding from Fight for Sight and The Worshipful Company of Spectacle Makers. P.G.H. is the recipient of a Fight for Sight ECI award. Funding to pay the Open Access publication charges for this article was provided by the Wellcome Trust.

## Supplementary Material

Supplementary Data

## Appendix

Tin Aung^1,2^, Ching-Yu Cheng^1,2,3^, Brian W Fleck^4^, Jane Gibson^5^, Alex W Hewitt^6^, Albert Hofman^7,8^, René Höhn^9^, Jost B Jonas^10^, Chiea-Cheun Khor^11^, Caroline CW Klaver^12^, Hans G Lemij^13^, Jiemin Liao^2^, Andrew J Lotery^14^, Yi Lu^15^, Stuart Macgregor^15^, Paul Mitchell^16,17^, Wishal D Ramdas^12^, Henriët Springelkamp^12^, E-Shyong Tai^3,18^, Yik-Ying Teo^3^, André G Uitterlinden^8,19^, Cornelia M van Duijn^7^, Leonieke ME van Koolwijk^8^, Johannes R Vingerling^12^, Veronique Vitart^20^, Eranga Vithana^1^, Jie Jin Wang^6,16^, Katie M Williams^21^, Robert Wojciechowski^22,23,24^, Tien-Yin Wong^1,2^, WTCCC2, Liang Xu^25^, Ekaterina Yonova-Doing^21^, Tanja Zeller^26^

^1^Singapore Eye Research Institute, Singapore National Eye Centre, Singapore; ^2^Department of Ophthalmology, National University of Singapore and National University Health System, Singapore; ^3^Saw Swee Hock School of Public Health, National University of Singapore and National University Health System, Singapore; ^4^Princess Alexandra Eye Pavilion, Edinburgh, UK; ^5^Genetic Epidemiology and Genomic Informatics Group, Human Genetics, Faculty of Medicine, University of Southampton, Southampton General Hospital, Southampton, UK; ^6^Centre for Eye Research Australia, University of Melbourne, Royal Victorian Eye and Ear Hospital, Melbourne, Australia; ^7^Department of Epidemiology, Erasmus Medical Center, Rotterdam, The Netherlands; ^8^Netherlands Consortium for Healthy Ageing, The Netherlands Genomics Initiative, Hague, The Netherlands; ^9^Department of Ophthalmology, University Medical Center Mainz, Mainz, Germany; ^10^Department of Ophthalmology, Medical Faculty Mannheim of the Ruprecht-Karls-University of Heidelberg, Mannheim, Germany; ^11^Division of Human Genetics, Genome Institute of Singapore, Singapore; ^12^Department of Epidemiology and Department of Ophthalmology, Erasmus Medical Center, Rotterdam, The Netherlands; ^13^Glaucoma Service, The Rotterdam Eye Hospital, Rotterdam, The Netherlands; ^14^Clinical Neurosciences Research Grouping, Clinical and Experimental Sciences, Faculty of Medicine, University of Southampton, Southampton General Hospital, Southampton, UK; ^15^Statistical Genetics, Queensland Institute of Medical Research, Brisbane 4029, Australia; ^16^Department of Ophthalmology, University of Sydney Centre for Vision Research, Sydney, NSW, Australia; ^17^Department of Ophthalmology, University of Sydney, Sydney, NSW, Australia; ^18^Department of Medicine, National University of Singapore and National University Health System, Singapore; ^19^Department of Epidemiology and Department of Internal Medicine, Erasmus Medical Center, Rotterdam, the Netherlands; ^20^MRC Human Genetics Unit, IGMM, University of Edinburgh, Edinburgh EH4 2XU, UK; ^21^Department of Twin Research and Genetic Epidemiology, King's College London, St. Thomas’ Hospital, London, UK; ^22^Department of Epidemiology, Johns Hopkins Bloomberg School of Public Health, Baltimore, MD, USA; ^23^Wilmer Eye Institute, Johns Hopkins School of Medicine, Baltimore, MD, USA; ^24^National Human Genome Research Institute (NIH), Baltimore, MD, USA; ^25^Beijing Institute of Ophthalmology, Beijing Tongren Hospital, Capital University of Medical Science, Beijing, China; ^26^Clinic for General and Interventional Cardiology, University Heart Center Hamburg, Hamburg, Germany.
