# Supplementary material for: A genome-wide association study of intra-ocular pressure suggests a novel association in the gene FAM125B in the TwinsUK cohort
Source: Hum Mol Genet. 2014 Feb 11;23(12):3343–8. doi: 10.1093/hmg/ddu050 (PMC4030784; doi:10.1093/hmg/ddu050)
Supplement: Supplementary Data [file supp_23_12_3343__index.html]

A genome-wide association study of intra-ocular pressure suggests a novel association in the gene FAM125B in the TwinsUK cohort — A genome-wide association study of intra-ocular pressure suggests a novel association in the gene FAM125B in the TwinsUK cohort — Supplementary Data 

# A genome-wide association study of intra-ocular pressure suggests a novel association in the gene *FAM125B* in the TwinsUK cohort

## Supplementary Data

Supplementary Data

**Files in this Data Supplement:**

- Supplementary Data - Docx file
