# Supplementary material for: A genome-wide association study of intra-ocular pressure suggests a novel association in the gene FAM125B in the TwinsUK cohort
Source: Hum Mol Genet. 2014 Feb 11;23(12):3343–8. doi: 10.1093/hmg/ddu050 (PMC4030784; doi:10.1093/hmg/ddu050)
Supplement: Supplementary Data [file supp_ddu050_ddu050supp.docx]

**Supplementary Information Page**

**Figure S1:** Q-Q plot of TwinsUK cohort (discovery study) 2

**Figure S2:** Forest plot for rs2286885 for the discovery 3

(TwinsUK) and replication cohorts

**Table S1:** Study characteristics of the TwinsUK cohort 4

**Table S2:** Results for rs2286885 in Asian cohorts of IGGC 4

Study descriptive, phenotyping methods and genotyping 5

Acknowledgements 13


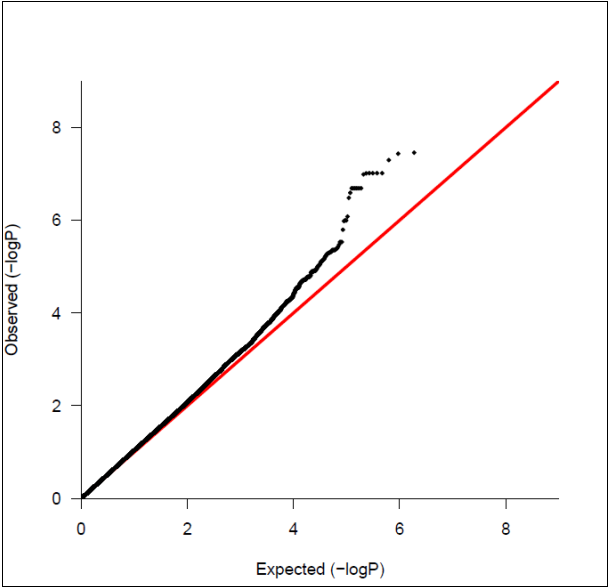
**Figure S1: A Q-Q plot of the observed versus expected p-values in the TwinsUK cohort**

**
 Figure S2: Forest plot for rs2286885 for the discovery (TwinsUK) and replication
 cohorts.**
 (ES = effect size; CI = Confidence intervals; BATS = Brisbane Adolescent Twin Study;
 BMES = Blue Mountains Eye Study; ERF = Erasmus Rucphen Family Study; GHS =
 Gutenberg Health Study; ORCADES = Orkney Complex Disease Study; RS = Rotterdam
 Study; TEST = Tasmanian Eye Study of Twins)

**Table S1:** **Study characteristics of the TwinsUK cohort**

|  | **IOP (mmHg)** | **age (years)** | **CCT (µm)** | **SBP (mmHg)** |
| --- | --- | --- | --- | --- |
| **Number of participants data available for** | 2774 | 2774 | 2555 | 2708 |
| **Mean** | 15.6 | 56.5 | 545 | 126.1 |
| **S.D. (range)** | 3.3 (7-38) | 11.8 (16-83) | 36.4 (211-657) | 16.6 (87-197) |

**Table S2: Results for rs2286885 in Asian cohorts of IGGC**

| **Cohort** | **Number of subjects*** | **Frequency^¶^** | **beta^¶^** | **SE** | **p-value** |
| --- | --- | --- | --- | --- | --- |
| BES | 805 | 0.35 | -0.105 | 0.158 | 0.507 |
| SCES | 1867 | 0.29 | -0.245 | 0.113 | 0.031 |
| SIMES | 2530 | 0.21 | -0.017 | 0.119 | 0.890 |
| SINDI | 2522 | 0.38 | 0.053 | 0.081 | 0.516 |

*Number of subjects refers to those with genotype information for rs2286885
 **^¶^** Frequency and beta refer to the frequency and the effect size respectively for the A
 allele of rs2286885
 BES = Beijing Eye Study; SCES = Singapore Chinese Eye Study;
 SIMES = Singapore Malay Eye Study; SINDI = Singapore Indian Eye Study.

**Study descriptives, phenotyping methods and genotyping**

**(A) Study descriptives**

**Blue Mountains Eye Study (BMES)**

Participants are part of the Blue Mountains Eye Study (BMES), a population-based eye disease survey in individuals living in the Blue Mountains region, west of Sydney, Australia.

Samples were genotyped on the Human660W-Quad. Imputation was performed with IMPUTE2 which adopts a two-stage approach using both haploid and diploid reference panels. 1000 Genome pilot data was used as reference panel. The SNPs considered in this study were those present also in HapMap2.

Intraocular pressure (IOP) was measured by applanation tonometry using a Goldmann tonometer (Haag-Streit, Bern, Switzerland). Vertical disc and cup diameter were obtained after pupil dilation from 30º colour stereoscopic optic disc photographs taken with a 99 Zeiss FF3 fundus camera (Carl Zeiss Meditec, Dublin, CA). Vertical cup-to-disc ratio (VCDR) was calculated by the vertical disc and cup measurements. Central corneal thickness, CCT, was measured using ultrasonic pachymetry.

The number of participants in the Blue Mountain Eye Study in the baseline is 3654 (57%males, 43% females), 2303 of them have imputed genotyping data available. The age range is 49-97 with mean 66.19 (±9.77) Intraocular pressure data and GWASs data are available for 1667 individuals with mean 16.04 mmHg (±2.63) and range 8.00-34.50 mmHg. 961 individuals have both IOP and CCT available. 1672 of the people with IOP have blood pressure measurements available. Information on IOP-lowering therapies and surgery for glaucoma are accessible and 10 individuals were deleted for ocular surgery.

**Erasmus Rucphen Family Study (ERF)**

The Erasmus Rucphen Family (ERF) Study is a family‐based cohort in a genetically isolated population in the southwest of the Netherlands with over 3,000 participants aged between 18 and 86 years^[[1]](#endnote-1),^^[[2]](#endnote-2)^. Cross‐sectional examination took place between 2002 and 2005. The IOP was measured with Goldmann applanation tonometry (Haag-Streit, Bern, Switzerland). IOP was measured twice per eye. If the two measurements in one eye differed, a third measurement was performed, and the median value was recorded. All measurements in these studies were conducted after the Medical Ethics Committee of the Erasmus University had approved the study protocols and all participants had given a written informed consent in accordance with the Declaration of Helsinki. DNA was genotyped on one of four different platforms (Illumina 6k, Illumina 318K, Illumina 370K and Affymetrix 250K), which were then merged. Samples with low call rate (<97.5%), with excess autosomal heterozygosity (>0.336), or with sex‐mismatch were excluded. A set of genotyped input SNPs with call rate >98%, with minor allele frequency >0.01, and with Hardy‐Weinberg P value >10^−6^ was used for imputation. We used the Markov Chain Haplotyping (MACH) package version 1.0.15 software (Rotterdam, The Netherlands; imputed to plus strand of NCBI build 36, HapMap release #22) for the analyses. For each imputed SNP, a reliability of imputation was estimated as the ratio of the empirically observed dosage variance to the expected binomial dosage variance (O/E ratio). GWAS analyses were performed using the ProbABEL package. Mmscore models were used to correct for family structure.

**Framingham Eye Study**

The Framingham Eye Study (FES) was nested within the Framingham Heart Study (FHS, http://www.framinghamheartstudy.org), which began its first round of extensive physical examinations in 1948 by recruiting 5,209 men and women from the town of Framingham, MA, USA. Surviving participants from the original cohort returned for biennial exams, which continue to the present. A total of 2675 FHS participants were also examined as part of the FES between 1973 and 1975. The FES was designed to evaluate ocular characteristics of examinees such as: senile cataract; age‐related macular disease; glaucoma; and retinopathy. Between 1989 and 1991, 1603 offspring of original cohort participants also received ocular examinations. The analyses in the current study are limited 1497 (42.5% men) participants from both the original and the offspring cohorts for whom genotype data were available. Most individuals in this analysis set are unrelated but a small number of related pairs remain. All data‐‐including refractive error, demographics and genotypes‐‐were retrieved from the database of Genotypes and Phenotypes (dbGaP) after approval for controlled access to individual‐level data. All study protocols are in compliance with the World Medical Association Declaration of Helsinki. Since 1971, written consent has been obtained from participants before each examination. The research protocols of the Framingham Heart Study are reviewed annually by the Institutional Review Board of the Boston University Medical Center and by the Observational Studies Monitoring Board of the National Heart, Lung and Blood Institute. Genotyping was conducted as part of the NHLBI Framingham SNP Health Association Resource (SHARe). This sub‐study contains genotype data for approximately 550000 SNPs (Affymetrix 500K mapping arrays [Mapping250k_Nsp and Mapping250K_Sty] plus Affymetrix 50K supplemental human gene‐focused array) in over 9200 FHS participants (1497 of whom were used in this analysis). Samples were chosen based on pedigree information and genotyping quality; Samples with a genotypic call rate below 95% were not chosen for analysis. The mean call rate for analyzed samples was 99.2% (SD=0.4%). Genotype data cleaning was carried‐out in several steps. The final marker list contained 436,494 high‐quality SNPs with a minor‐allele frequency >= 0.01, a Mendelian error rate below 2% across all pedigrees, a genotype call rate above 95%, and whose distribution was consistent with Hardy‐Weinberg expectations (P>0.0001). Genotype imputation to the HapMap‐II reference panel (CEU population release 22, NCBI build 36) was carried out in a two‐step process using the Markov Chain Haplotyping (MACH version 1.0.16.a) software. First, crossover and error‐rate maps were built using 400 unrelated individuals (200 male and 200 female) sampled from FHS subjects. Second, genotype imputations of approximately 2.5 million autosomal HapMap‐II SNPs were carried out on the entire FHS dataset using parameters estimated from step 1.

**Gutenberg Health Study (GHS1, GHS2)**

The Gutenberg Health Study (GHS) is a population‐based, prospective, observational cohort study in the Rhine‐Main Region in Midwestern Germany with a total of 15,010 participants and follow‐up after five years. The study sample is recruited from subjects aged between 35 and 74 years at the time of the exam. The sample was drawn randomly from local governmental registry offices and stratified by gender, residence (urban and rural) and decade of age. Exclusion criteria were insufficient knowledge of the German language to understand explanations and instructions, and physical or psychic inability to participate in the examinations in the study center. The study design comprises an ophthalmological examination, general and especially cardiovascular examinations, psychosomatic evaluation, laboratory tests, and biobanking for proteomic and genetic analyses. All participants underwent an ophthalmological investigation taking place between 11:00 a.m. and 8:00 p.m. IOP was measured with a non-contact automated air-puff controlled tonometer (Nidek NT-2000™, Nidek Co., Japan). Starting always with the right eye, the mean of three measurements within a 3 mm Hg range was obtained for each eye. Central corneal thickness was measured using Scheimpflug imaging provided with the Pachycam™ (Oculus, Wetzlar, Germany). Each participant received three standardized systolic blood pressure measurements (SBP) in sitting position using the Omron HEM 705-CP II (OMRON, Mannheim, Germany). The mean SBP from readings after 8 and 11 minutes of rest was considered for the analysis. The study was approved by the Medical Ethics Committee of the University Medical Center Mainz and by the local and federal data safety commissioners. According to the tenets of the Declaration of Helsinki, written informed consent was obtained from all participants prior to entering the study.

Within GHS, DNA was extracted from buffy-coats from EDTA blood samples. Genetic analysis was conducted in the first 5,000 study participants. For these, 3,463 individuals were genotyped in 2008 (GHS1) and further 1,439 individuals in 2009 (GHS2). Genotyping was performed for GHS1 and GHS2 using the Affymetrix Genome-Wide Human SNP Array 6.0 (<http://www>.affymetrix.com), as described by the Affymetrix user manual. Genotypes were called using the Affymetrix Birdseed-V2 calling algorithm. Individuals with a call rate below 97% or a too high autosomal heterozygosity (3 s.d. from mean) and sex-mismatches were excluded. After applying standard quality criteria (minor allele frequency >1%, genotype call rate >98% and P-value of deviation from Hardy-Weinberg equilibrium of >0.0001), 675,350 SNPs in 2750 individuals from GHS1 and 673,914 SNPs in 1,143 individuals from GHS2 remained for analysis. Imputation of missing genotypes was performed using Impute software v2.1.0 and HapMap release 24, NCBI Build 36.

**Orkney Complex Disease Study (ORCADES)**

The Orkney Complex Disease Study (ORCADES) is a population‐based, cross‐sectional study in the Scottish archipelago of Orkney, including 1,285 individuals with eye measurements. The study received approval from relevant ethics committees in Scotland and followed the tenets of the Declaration of Helsinki.

IOP was measured with a tonopen.

Measures on eyes with a history of trauma were removed and the analysis was done on the average of both eye measures, or on one eye measure only when the fellow-eye measurement was missing. 474 individuals which had been genotyped and had IOP measurements were used in this analysis. Genome-wide association analysis was performed using the “mmscore” function of ProbABEL (<http://www.genabel.org/packages>) under an additive model for the SNP allelic effect. This score test for family based association takes into account relationship structure and allowed unbiased estimations of SNP allelic effect when relatedness is present between examinees. The relationship matrix used in this analysis was generated by the “ibs” function of GenABEL (using weight= “freq” option), which uses genomic data to estimate the realized pair-wise kinship coefficients.

Genotypes were generated using HumanHap 300v2 or 370CNV-Quad Illumina SNP arrays. Genotypes were determined using the Illumina BeadStudio software following the manufacturer’s standard recommendations. Samples with a call rate below 97 % (for SNPs with call rates above 98%, a minor allele frequency below 1% and p-value for Fisher’s exact test of Hardy Weinberg equilibrium (HWE)>10-6), potentially mixed samples with excess autosomal heterozygosity or gender discrepancy (based on the genotyped sex chromosomes), and ethnic outliers (based on principal components analysis of genotypic data), were excluded from the analysis using the quality control algorithm implemented in GenABEL. Imputation of allele dosage for over 2 millions SNPs on the 22 autosomal chromosomes with reference to HapMap CEU build 36 release 22 was performed using the software MACH v1.0.15 and allele doses used in the linear additive model for testing association.

**Rotterdam Study (RS-I, RS-II, RS-III)**

The Rotterdam Study is a prospective population‐based cohort study in the elderly living in Ommoord, a suburb of Rotterdam, the Netherlands^[[3]](#endnote-3)^. In brief, the Rotterdam Study consists of 3 independent cohorts: RS-I, RS-II, and RS-III. Participants underwent multiple physical examinations with regular intervals from 1991 to present. The IOP was measured with Goldmann applanation tonometry (Haag-Streit, Bern, Switzerland). IOP was measured twice per eye. If the two measurements in one eye differed, a third measurement was performed, and the median value was recorded. All measurements in RS‐I, RS-II and RS-III were conducted after the Medical Ethics Committee of the Erasmus University had approved the study protocols and all participants had given a written informed consent in accordance with the Declaration of Helsinki. DNA was extracted from blood leucocytes according to standard procedures. Genotyping of SNPs was performed using the Illumina Infinium II HumanHap550 chip v3.0 array (RS‐I); the HumanHap550 Duo Arrays and the Illumina Human610‐Quad Arrays (RS‐II), and the Human 610 Quad Arrays Illumina (RS‐III). Samples with low call rate (<97.5%), with excess autosomal heterozygosity (>0.336), or with sex‐mismatch were excluded, as were outliers identified by the identity‐by‐state clustering analysis (outliers were defined as being >3 s.d. from population mean or having identity‐by‐state probabilities >97%). We used genomic control to obtain optimal and unbiased results and applied the inverse variance method of each effect size estimated for both autosomal SNPs that were genotyped and imputed in both cohorts. A set of genotyped input SNPs with call rate >98%, with minor allele frequency >0.01, and with Hardy‐Weinberg P value >10^−6^ was used for imputation. We used the Markov Chain Haplotyping (MACH) package version 1.0.15 software (Rotterdam, The Netherlands; imputed to plus strand of NCBI build 36, HapMap release #22) for the analyses. For each imputed SNP, a reliability of imputation was estimated as the ratio of the empirically observed dosage variance to the expected binomial dosage variance (O/E ratio). GWAS analyses were performed using GRIMP.

**Southampton cohort**

These data were created and analysed as a case/control GWAS. These were pre-selected to exclude; 1) pseudoexfoliation cases, 2) myocilin mutation positive cases. Primary open angle glaucoma patients (n=400) were genotyped on the Affymetrix SNP 6.0 array, all data were exported on the forward strand. These data were compared with the Affymetrix SNP 6.0 data publically available for the WTCCC2 controls.

Quality control steps involved removing cases and SNPs with a high degree of missingness (>10%), and removing SNPs with a minor allele frequency less than 5%. We also carried out identity by state (IBS) analysis to identify unknown relatives or duplicates and multi-dimensional scaling (MDS) to identify those with differing ethnic backgrounds to the majority of the group (a Caucasian cohort). 387 cases were available for analysis after this step. After also removing cases that have undergone trabeculectomy or cataract surgery, 166 cases remain for analysis.

The HWE test (in controls) is generally used to control for genotyping error when cases and controls are genotyped together. However as the cases were genotyped separately extra QC steps were carried out. Firstly, the average confidence score for the genotypes (from the Affymetrix SNP calling software “Genotyping console”) were calculated for each SNP across individuals and SNPs with confidence scores which fell two standard deviations above the mean (worse confidence) were removed. We also removed SNPs with extreme deviations from HWE (p<1x10^-10^) in cases. Three known tri-allelic SNPs were also removed. 681,549 SNPs were available for analysis after QC.

HapMap phase 2, release 22, build 36 (forward strand) data on 60 CEU samples were used as the reference for imputation. This was carried out in PLINK and after imputation a total of 2,340,809 SNPs were available for analysis.

**Tasmanian Eye Study of Twins/ Brisbane Adolescent Twin Study (TEST/BATS)**

The Australian Twin Eye Study comprises participants examined as part of the Twins Eye Study in Tasmania or the Brisbane Adolescent Twins Study. In most participants, the IOP was measured with the TONO-PEN XL (Reichert, Inc. New York, USA). The Australian cohorts were genotyped on the Illumina Human Hap610W Quad array, with part of the sample typed alongside the TwinsUK cohort and the remainder typed as a separate contract with DeCODE genetics. The inclusion criteria for the SNPs were a minor allele frequency >0.01, Hardy-Weinberg equilibrium p≥10^-6^, and a SNP call rate >95% or Illumina Beadstudio Gencall Score ≥0.7, resulting in 543,862 SNPs. Imputation was done with reference to HapMap release 22 CEU using MACH (<http://www.sph.umich.edu/csg/abecasis/MACH/>). Data from 1815 people, from 867 families, were included in the analyses. The mean IOP of both eyes was used as outcome variable. Association analyses were performed in Merlin (<http://www.sph.umich.edu/csg/abecasis/merlin/>) by using the –fastassoc option. Age, sex and measurement technique (tonopen or Goldmann applanation tonometry) were fitted as covariates. Ancestry, initially determined through self-reporting, was verified through Principal Component decomposition.

**(B) Phenotyping method**

| **Study** | **IOP measurment method** |
| --- | --- |
| BMES | Goldmann applanation tonometry (Haag-Streit, Bern, Switzerland), |
| ERF | Goldmann applanation tonometry (Haag-Streit, Bern, Switzerland) |
| Framingham | Goldmann applanation tonometry (Haag-Streit, Bern, Switzerland) |
| GHSI | ORA (Nidek NT-2000™, Nidek Co., Japan) |
| GHSII | ORA (Nidek NT-2000™, Nidek Co., Japan) |
| ORCADES | TONO-PEN XL (Reichert, Inc. New York, USA) |
| RSI | Goldmann applanation tonometry (Haag-Streit, Bern, Switzerland) |
| RSII | Goldmann applanation tonometry (Haag-Streit, Bern, Switzerland) |
| RSIII | Goldmann applanation tonometry (Haag-Streit, Bern, Switzerland) |
| Southampton | Goldmann applanation tonometry (Haag-Streit, Bern, Switzerland) |
| TEST/BATS | TONO-PEN XL (Reichert, Inc. New York, USA) |

**(C) Genotyping method**

| **Study** | **Genotyping Chip** |
| --- | --- |
| BMES | Human660W-Quad array |
| ERF | Illumina 6k; Illumina 318K; Illumina 370K; Affymetrix 250K |
| Framingham | AFFY HuGeneFocused_50K; AFFY Mapping250k_Nsp; and AFFY Mapping250K_Sty |
| GHSI | Affymetrix Genome‐Wide Human SNP 6.0 Array |
| GHSII | Affymetrix Genome‐Wide Human SNP 6.0 Array |
| ORCADES | Illumina HumanHap 300v2; 370CNV‐Quad |
| RSI | Illumina Infinium II HumanHap550 chip v3.0 array |
| RSII | HumanHap550 Duo Arrays + Human610‐Quad Arrays Illumina |
| RSIII | Human 610 Quad Arrays Illumina |
| Southampton | Affymetrix SNP 6.0 array |
| TEST/BATS | Illumina HumanHap 610W Quad arrays (Illumina Inc., San Diego, CA, USA) |

**Acknowledgements**

**Beijing Eye Study (BES)** was supported by National Natural Science Foundation of China (grant 81170890).

**BMES** was supported by the Australian National Health & Medical Research Council (NH&MRC), Canberra Australia (974159, 211069, 457349, 512423, 475604, 529912); the Centre for Clinical Research Excellence in Translational Clinical Research in Eye Diseases; NH&MRC research fellowships (358702, 632909 to J.J.W, 1028444 to P.N.B.); and the Wellcome Trust, UK as part of Wellcome Trust Case Control Consortium 2 (A. Viswanathan, P. McGuffin, P. Mitchell, F. Topouzis, P. Foster) for genotyping costs of the entire BMES population (085475B08Z, 08547508Z, 076113).

The Centre for Eye Research Australia receives Operational Infrastructure Support from the Victorian government. BMES acknowledges Elena Rochtchina from the Centre for Vision Research, Department of Ophthalmology and Westmead Millennium Institute University of Sydney (NSW Australia); John Attia, Rodney Scott, Elizabeth G. Holliday from the University of Newcastle (Newcastle, NSW Australia); Jing Xie, and Andrea J. Richardson from the Centre for Eye Research Australia, University of Melbourne; Michael T. Inouye, Medical Systems Biology, Department of Pathology & Department of Microbiology & Immunology, University of Melbourne (Victoria, Australia); Ananth Viswanathan, Moorfields Eye Hospital (London, UK); Paul J. Foster, NIHR Biomedical Research Centre for Ophthalmology, UCL Institute of Ophthalmology & Moorfields Eye Hospital (London); Peter McGuffin, MRC Social Genetic and Developmental Psychiatry Research Centre, Institute of Psychiatry, King's College (London, United Kingdom); Fotis Topouzis, Department of Ophthalmology, School of Medicine, Aristotle University of Thessaloniki, AHEPA Hospital (Thessaloniki, Greece); Xueling Sim, National University of Singapore.

**Framingham Eye Study** was supported by NEI (N01EY22112, N01EY92109); NEI Mentored Clinical Scientist Research Career Development Award (1K08EY022943‐01 to R.W.); the National Heart, Lung, and Blood Institute (N02HL64278) for SHARe genotyping; Boston University (N01HC25195); and by intramural funds of the National Human Genome Research Institute, NIH, USA (to R.W. and J.E.B.W.).

**Gutenberg Health Study** was funded through the government of Rheinland‐Pfalz ("Stiftung Rheinland Pfalz für Innovation" (AZ961386261733); the research programs "Wissen schafft Zukunft" and "Schwerpunkt Vaskuläre Prävention" of the Johannes Gutenberg‐University of Mainz; Boehringer Ingelheim; PHILIPS Medical Systems; National Genome Network ''NGFNplus'' by the Federal Ministry of Education and Research, Germany (A301GS0833). GHS acknowlegdes Dagmar Laubert‐Reh for data analysis.

**ORCADES** was supported by the Chief Scientist Office of the Scottish Government, the Royal Society, the Medical Research Council Human Genetics Unit and the European Union framework program 6 EUROSPAN project (LSHGCT2006018947). ORCADES acknowledges the invaluable contributions of Lorraine Anderson and the research nurses in Orkney, in particular Margaret Pratt who performed the eye measurements,as well as the administrative team in Edinburgh University; and the Wellcome Trust Clinical facility (Edinburgh, United Kingdom) for DNA extraction; and Peter Lichner and the Helmholtz Zentrum Munchen (Munich, Germany) for genotyping; and Mirna Kirin for the genetic data imputation.

**Rotterdam Study** and **ERF** were supported by the Netherlands Organisation of Scientific Research (NWO); Erasmus Medical Center and Erasmus University, Rotterdam, The Netherlands; Netherlands Organization for Health Research and Development (ZonMw); UitZicht; the Research Institute for Diseases in the Elderly; the Ministry of Education, Culture and Science; the Ministry for Health, Welfare and Sports; the European Commission (DG XII); the Municipality of Rotterdam; the Netherlands Genomics Initiative/NWO; Center for Medical Systems Biology of NGI; Stichting Lijf en Leven; Stichting Oogfonds Nederland; Landelijke Stichting voor Blinden en Slechtzienden; Algemene Nederlandse Vereniging ter Voorkoming van Blindheid; Medical Workshop; Heidelberg Engineering; Topcon Europe BV. We acknowledge the contribution of Ada Hooghart, Corina Brussee, Riet Bernaerts‐Biskop, Patricia van Hilten, Pascal Arp, Jeanette Vergeer, Maarten Kooijman and Lennart Karssen.

The generation and management of GWAS genotype data for the Rotterdam Study is supported by the Netherlands Organisation of Scientific Research NWO Investments (nr. 175.010.2005.011, 911-03-012). This study is funded by the Research Institute for Diseases in the Elderly (014-93-015; RIDE2), the Netherlands Genomics Initiative (NGI)/Netherlands Organisation for Scientific Research (NWO) project nr. 050-060-810. We thank Pascal Arp, Mila Jhamai, Marijn Verkerk, Lizbeth Herrera and Marjolein Peters for their help in creating the GWAS database, and Karol Estrada and Maksim V. Struchalin for their support in creation and analysis of imputed data.

The authors are grateful to the study participants, the staff from the Rotterdam Study and the participating general practitioners and pharmacists.

**Singapore Chinese Eye Study (SCES), Singapore Malay Eye Study (SiMES)** and **Singapore Indian Eye Study (SINDI)** were supported by the National Medical Research Council (NMRC), Singapore (grants 0796/2003, IRG07nov013, IRG09nov014, NMRC 1176/2008, STaR/0003/2008, CG/SERI/2010), and Biomedical Research Council (BMRC), Singapore (08/1/35/19/550 and 09/1/35/19/616). The Singapore Tissue Network and the Genome Institute of Singapore, Agency for Science, Technology and Research, Singapore provided services for tissue archival and genotyping, respectively.

**Southampton cohort**

We thank Marie Nelson, Catrin Watkins, Georgina Matei, and the Southampton Wellcome Trust Clinical Research Facility for research nurse support in collecting DNA samples and all the patients who contributed to this work. Funding for this work was provided by: Optegra, UK and Eire Glaucoma Society, T F C Frost Charitable Trust.

**TEST** and **BATS** (Australian Twins) were supported by an Australian National Health and Medical Research Council (NHMRC) Enabling Grant (2004‐2009, 350415, 2005‐2007); Clifford Craig Medical Research Trust; Ophthalmic Research Institute of Australia; American Health Assistance Foundation; Peggy and Leslie Cranbourne Foundation; Foundation for Children; Jack Brockhoff Foundation; National Institutes of Health/National Eye Institute (RO1EY01824601 (2007‐2010)); Pfizer Australia Senior Research Fellowship (to D.A.M.); and Australian NHMRC Career Development Award (to S.M.). Genotyping was funded by an NHMRC Medical Genomics Grant; US National Institutes of Health/National Eye Institute (1RO1EY018246), Australian sample imputation analyses were carried out on the Genetic Cluster Computer which is financially supported by the Netherlands Scientific Organization (NWO48005003). Australian Twins thanks Nicholas Martin, Scott Gordon, Dale Nyholt, Sarah Medland, Brian McEvoy, Margaret Wright, Anjali Henders, Megan Campbell for ascertaining and processing genotyping data; Jane MacKinnon, Shayne Brown, Lisa Kearns, Jonathan Ruddle, Paul Sanfilippo, Sandra Staffieri, Olivia Bigault, Colleen Wilkinson, Jamie Craig, Yaling Ma, Julie Barbour for assisting with clinical examinations; and Dr Camilla Day and staff at the Center for Inherited Disease Research.

TwinsUK was funded by the Wellcome Trust; European Community’s Seventh Framework Programme (FP7/2007-2013). The study also receives support from the National Institute for Health Research (NIHR) BioResource Clinical Research Facility and Biomedical Research Centre based at Guy's and St Thomas' NHS Foundation Trust and King's College London. Tim Spector is holder of an ERC Advanced Principal Investigator award. SNP Genotyping was performed by The Wellcome Trust Sanger Institute and National Eye Institute via NIH/CIDR. A.N. received funding from Fight for Sight and The Worshipful Company of Spectacle Makers. P.G.H. is the recipient of a Fight for Sight ECI award. C.J.H. is an NIHR Senior Research fellow. We acknowledge the contribution of Drs Toby Andrew, Margarida Lopes, Samantha Fahy and Diana Kozareva.

1. Aulchenko, Y.S. et al. (2004) Linkage disequilibrium in young genetically isolated Dutch population . Eur J Hum Genet, **12**, 527‐534. [↑](#endnote-ref-1)
2. Pardo, L.M., MacKay, I., Oostra, B., van Duijn, C.M. & Aulchenko, Y.S. (2005) The effect of genetic drift in a young genetically isolated population. Ann Hum Genet, **69**, 288‐295. [↑](#endnote-ref-2)
3. Hofman, A. et al. (2011) The Rotterdam Study: 2012 objectives and design update. Eur J Epidemiol, **26**, 657‐686. [↑](#endnote-ref-3)
